# HRQOLISP-26: A Concise, Multiculturally Valid, Multidimensional, Flexible, and Reliable Stroke-Specific Measure

**DOI:** 10.5402/2011/295096

**Published:** 2011-12-06

**Authors:** Mayowa Ojo Owolabi

**Affiliations:** Department of Medicine, University College Hospital, PMB 5116, Ibadan 200001, Nigeria

## Abstract

*Background*. A multidimensional, brief, and flexible stroke-specific health-related quality of life (HRQOL) measure is still needed. The aim was to develop a shortened version of the HRQOLISP-102, a multiculturally generated measure with excellent psychometric properties. *Methods*. Participants included 100 (Ibadan, Nigeria) and 103 (Berlin, Germany) stroke patients compared to 100 (Ibadan) and 50 (Berlin) apparently healthy adults. Using standard protocol, the 26-item version was generated, consisting of therapeutically relevant physical, psychological, cognitive, and ecosocial domains. Criterion validity of the HRQOLISP-26 was determined using Bland-Altman statistics. “Known groups” validity was assessed using NIHSS, stroke levity score, and modified Rankin scale. *Results*. HRQOLISP-26 was easily interpretable and precise with no significant floor/ceiling effect. It can be completed within 7 minutes. It showed good content, construct, “known groups,” and criterion validity. It demonstrated good internal consistency (*α* = 0.81, 0.89) and test-retest reliability. *Conclusions*. HRQOLISP-26 is novel, brief, multiculturally-valid, and flexible for routine assessment of HRQOL in stroke patients.

## 1. Introduction

The number of stroke patients is increasing particularly in low- and medium-income countries [1, 2]. The ultimate goal of rehabilitation of stroke patients is to improve their quality of life. Health-related quality of life (HRQOL) measures are therefore crucial for the routine evaluation of the patient's rehabilitation needs, prognosis, and response to various therapies (including physical, psychological, cognitive, and occupational therapies).

The 102-item HRQOL in stroke patients (HRQOLISP) is a multiculturally generated, multifaceted, well-validated, holistic measure with excellent psychometric properties [3–5]. It consists of two spheres with seven domains. Its physical sphere comprises the therapeutically relevant physical, psychoemotional, cognitive and ecosocial domains. However, its length may make it unattractive for routine clinical use. 

Short versions of other HRQOL measures, including the SSQOL-12, SIS-16, and QLASS-19, are brief but have inadequate content validity, due to exclusion of therapeutically relevant multidimensional structure central to the HRQOL concept [3, 6–9]. The SIS-16 assesses only the physical domain, the SSQOL-12 assesses only two dimensions [7] while the QLASS has no distinct cognitive and social domains [8].

 A shortened version of the HRQOLISP-102, the HRQOLISP-40, is designed for studies of internal adaptation and disability disparity in stroke patients [10, 11]. However, a shorter version still, is necessary for routine clinical use and evaluation of various interventions. Such a shorter version should be administrable within a few minutes so as to reduce respondent burden. However, it should remain sufficiently multidimensional to obviate the need for combination with several other instruments which would still constitute more burden for the patient. 

The aims of this study were to develop a flexible shorter version of the HRQOLISP suitable for regular assessment of all routinely crucial domains of HRQOL and to determine if its psychometric robustness, particularly its multidimensional construct and “known groups” validity, is retained despite further item reduction.

## 2. Methods

### 2.1. Design and Participants

In line with convention, data obtained from previous validation studies of the HRQOLISP were utilized [6, 7, 12]. This included 353 respondents (100 stroke patients and 100 apparently healthy adults [13, 14] in Ibadan; and 103 stroke and 50 controls in Berlin). As is done conventionally, apparently healthy controls were used for comparison as any other group of respondents will have impaired HRQOL [13, 14]. Furthermore, within-group comparison was conducted across stroke severity strata. Those excluded from the study were patients who had ambiguous diagnosis of stroke or other medical conditions that were neither risk factors for nor complications of stroke but could interfere with HRQOL. Ethical permission was obtained from the ethical committees of the University of Ibadan and Charite Universitaetmedizin Berlin.

### 2.2. Measures

Stroke levity scale (SLS) was applied as an index of stroke severity. The SLS correlates significantly to the NIHSS (rho = −0.79, *P* < 0.0001) and is a valid measure of stroke severity which can be used in illiterate populations [15]. Stroke was classified using the WHO stroke scale [16–19] and brain CT. Disability was assessed with the modified Rankin scale (mRS) while HRQOL was measured by the 102-item HRQOLISP instrument whose characteristics are described in detail elsewhere [5]. Assessments were made at least one month after stroke to consecutive stroke patients.

 The HRQOLISP has been shown to be both interviewer- and self-administrable with mode of administration having no significant impact on scores [11]. In Ibadan, a subset of stroke patients (20) had repeat interview within 2 days after the first assessment by the same interviewer. In Berlin, a reassessment was similarly conducted in 10 patients, within one week.

### 2.3. Data Analysis

Data analysis was carried out using gold standard methods widely reported in HRQOL literature [14, 20–22]. From data generated in both cities, using a combination of factor analysis (item-total correlation, item-factor loading), floor and ceiling effects, skewness, test-retest weighted-kappa statistics and item contribution to content and construct validity, the best 26 items were chosen out of a total of 63 initial items in the physical sphere [21] (Appendices  I and II). Thereafter the score for each HRQOLISP-26 and HRQOLISP-63 domain was generated by Likert's method which facilitates interpretation and interindividual comparisons. The domain scores were transformed into a scale with a maximum score of 100 (best health) each. The overall scores were generated by finding the arithmetic mean of the domain scores. 

### 2.4. A Priori Statements

To establish discriminant validity, stroke patients are expected to have worse HRQOL profile than healthy controls. To establish “known groups” validity, there should be significant differences in mean HRQOL scores across mRS, SLS and NIHSS strata. To establish criterion validity, there should be good agreement and strong (*ρ* ≥ 0.60) correlation between the corresponding domains and overall scores of the HRQOLISP-26 and HRQOLISP-63 [21].

### 2.5. Psychometric Analysis of HRQOLISP-26

*Discriminant validity* was assessed between the test and control populations using Student's *t*-test and ANCOVA. “Known groups” validity was evaluated by comparing responses from patients with various severities of stroke using Kruskal-Wallis test [20]. *Criterion validity* was assessed using correlation statistics and Bland-Altman plots comparing the HRQOLISP 26 to the HRQOLISP-63 as a gold standard [7, 23]. The percentages of variance of the HRQOLISP-63 scores that could be explained by the 26-item version scores were computed [7, 23]. At the individual level, the limits of agreement (±1.96 × SD_difference_) were calculated using the Bland-Altman procedure [7, 23]. Agreements between the corresponding domain and overall scores of the 26-item and 63-item versions were examined by computing the mean differences, the 95% confidence interval, and the effect sizes. Effect sizes were obtained by dividing the mean differences by the standard deviation (SD) of the corresponding 63-item HRQOLISP score [7]. The conventional interpretation of effect sizes is 0.2 is small, 0.5 is medium, and 0.8 is large [7, 23]. 

Internal consistency *reliability* was determined by calculating Cronbach's coefficients [20]. For test-retest reliability, weighted kappa statistics was calculated [24]. Floor effect was defined as the presence of greater than 20% of the respondents scoring <10% [21]. Ceiling effect was acknowledged if the percentage of those with domain score >90% was greater than 20% of the respondents [21]. The statistically significant two-tailed *P* value (alpha) was set at <0.05. Data was analyzed using the SPSS software.

## 3. Results

The characteristics of the respondents are presented in Table  S1 (see supplementary material available online at doi: 10.5402/2011/295096). They were drawn from many ethnic groups (including Yoruba, Igbo, Hausa) in Nigeria, a developing country, and several ethnic groups (including German, Turkish, Russian, Spanish, Korean) in Germany, an industrialized country. 

### 3.1. Development of the HRQOLISP-26

The best 26 items were chosen out of a total of 63 initial items in the physical sphere of the HRQOLISP-102. Most of the items had very strong item-domain correlation (0.70 to 0.90). Four items with moderate item-domain correlation but pivotal contribution to the content validity and test-retest reliability were retained. Given that in Germany and Nigeria, the 102-item version took about 27 minutes to complete, the estimated completion time of the HRQOLISP-26 is 7 minutes. It comprises four therapeutically relevant domains.

### 3.2. Criterion Validity of the HRQOLISP-26

**Figure 1:**
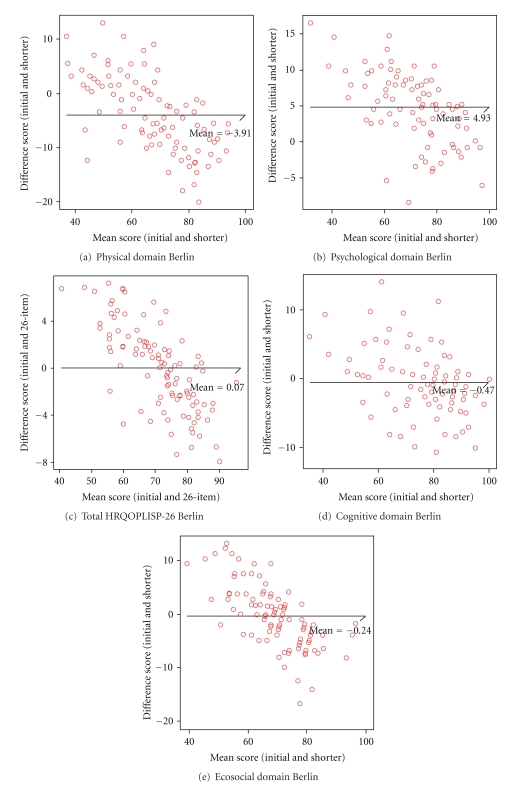
Bland-Altman plots of the HRQOLISP-26 versus HRQOLISP-63 (Berlin). Bland-Altman plots of the differences between the 63-item physical sphere of the HRQOLISP (*initial*) and the HRQOLISP-26 (*shorter*) scores related to the mean of the HRQOLISP-63 and HRQOLISP-26 scores for domains and total score in Berlin. The *x*-axes represent levels of HRQOL calculated as the means of the initial and shorter version scores and the *y*-axes represent the differences between scores on initial and shorter versions. The horizontal lines represent the mean difference.

In both countries, the HRQOLISP-26 domain and overall scores correlated strongly (rho = 0.90 to 0.97, *P* < 0.000001) to the corresponding HRQOLISP-63 scores and explained 82 to 95% of the variance of the long version (Table 1). The overall HRQOLISP-26 score explained 93% of the variance of the long version in Ibadan, and 95% in Berlin (Table 1). At the individual level, the mean differences between the HRQOLISP-26 and HRQOLISP-63 scores were not significant (Table 1). The limits of agreement were small (below 10%) with small-to-medium effect sizes (Table 1). The Bland-Altman plots (Figure 1 and Figure  S1) for the domain and total scores show that in both countries, the differences in individual scores were distributed evenly about the mean line across the full range of the mean scores.

 At the population level, the differences in mean scores between the short and the long versions were negligible (Table 1). The biggest difference was 5.4 on a scale of 0 to 100 in the domains, some of which had nearly identical scores. For the total scores, the differences were 2.3% in Ibadan, 0.1% in Berlin. The effect sizes of the differences were 0.23 for Ibadan and 0.01 for Berlin.

### 3.3. Construct and “Known Groups” Validity

**Figure 2:**
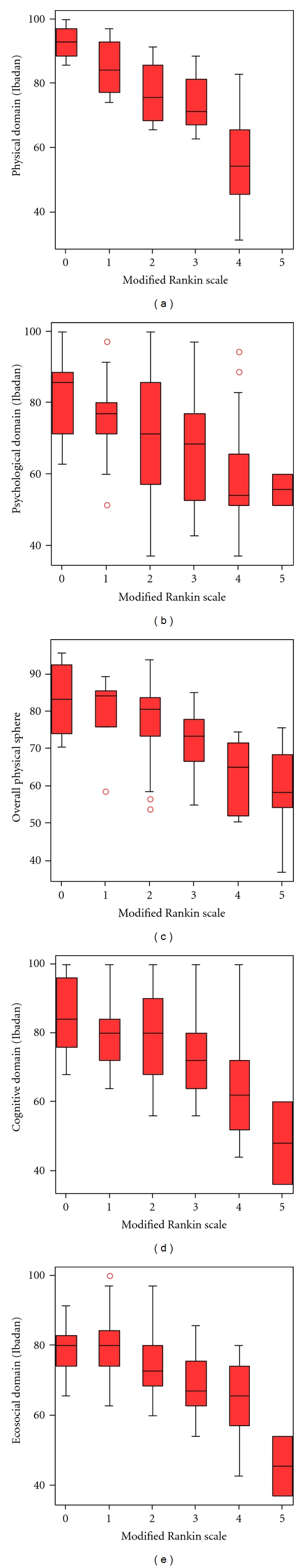
Box plot for HRQOLISP scores versus mRS strata in Ibadan.

The HRQOLISP-26 was able to discriminate between the stroke and control groups in all domains in both cities even after adjusting for possible confounders (0.0001 < *P* < 0.024,  Table 2). Across SLS and mRS strata in Ibadan as well as SLS, mRS, and NIHSS strata in Berlin, the HRQOLISP-26 showed significant “known groups” and construct validity in all domains (0.0001 < *P* < 0.042) except the cognitive domain in Berlin (Table 2, Figure 2 and Figure  S2). However, the cognitive domain demonstrated discriminant validity between stroke and control groups in Berlin and Ibadan, and “known groups” validity across NIHSS strata in Berlin (*P* = 0.034) and across SLS, mRS strata in Ibadan. The overall HRQOLISP-26 score demonstrated “known groups” and construct validity across SLS, mRS in both cities and NIHSS strata in Berlin (Table 2, Figure 2 and Figure  S2).

### 3.4. Reliability, Floor, and Ceiling Effects

Results of the KMO test showed satisfactory sampling adequacy (≥0.60) and Bartlett's test of sphericity was significant for all models. One-factor solution explained >50% of the variance for most domains in both cities (Table 3). Floor effect was absent in all domains in both countries. Ceiling effect was absent in all domains in Berlin and Ibadan except the physical domain in Ibadan (Table 3). However, only 2% scored 100% in the physical domain in Ibadan, while in both cities <2% scored 100% overall. The Cronbach's alpha was ≥0.7 in all domains in both cities (Table 3). It was 0.81 (Berlin) and 0.89 (Ibadan) for the total HRQOLISP-26 score.

## 4. Discussion

Despite previous research efforts, the need remains for a multidimensional but brief, patient-centered but therapeutically relevant, multiculturally-valid but easily interpretable, flexible but reliable stroke-specific HRQOL measure [3, 9, 25, 26]. Such a balance has not been meticulously attained by preexisting measures. Generic measures are not designed for specific diseases and are thus not sensitive to subtle patient-specific and disease-specific changes in HRQOL [3, 4, 27]. Furthermore, because they lack adequate content validity for stroke, generic measures are not suitable for routine clinical use or clinical trials in stroke [3, 4, 27]. 

To reduce respondent burden, improve acceptability, and routine utility, the trend is towards the development of concise measures. Examples of stroke-specific brief measures include the SIS-16, the SSQOL-12, and QLASS-19. While these measures are brief and user friendly, they sacrificed multidimensionality and content validity for brevity. Such paucidimensional measures are often combined in series to cover all relevant dimensions thus subjecting patients to an unacceptable burden which reduces the overall frequency and reliability of responses [28]. 

The HRQOLISP is the first multicultural-generated, holistic, patient-centred, and multidimensional stroke-specific measure [10, 29, 30]. The essence of this study was to shorten it without compromising its therapeutically relevant multidimensionality, content validity, and reliability. 

### 4.1. Content and Criterion Validity of the HRQOLISP-26

*Content validity *is an assessment of how well the domains of interest are sampled. The HRQOLISP-26 contains items recommended for HRQOL measures [31, 32] distributed in therapeutically relevant domains. The physical domain corresponds to physiotherapy, psychological domain to psychotherapy, cognitive domain to speech, language and cognitive therapy, and ecosocial domain to occupational therapy and social reintegration. However, the spiritual sphere covered in the HRQOLISP-40 and HRQOLISP-102 is excluded. Therefore, for studies of internal adaptation and disability disparity the HRQOLISP-40 or HRQOLISP-102 is better [10, 11].

In both countries and cultural settings, with negligible mean differences between the HRQOLISP-26 and HRQOLISP-63 domains and total scores, at the individual and population levels, the HRQOLISP-26 has good *criterion validity*. This is further corroborated by the very strong correlation and percentage explained variance, small effect sizes and limits of agreement, and the favourable Bland-Altman plots. Therefore, in multicultural settings, for all types of stroke, the HRQOLISP-26 has sufficient criterion validity to be used instead of the HRQOLISP-63 as a whole or for the assessment of the individual domains. 

Although the SSQOL-12 which has physical and psychosocial domains, has good criterion validity, it does not have distinct cognitive and ecosocial domains. Likewise the SIS-16 is dimension-specific, covering only the physical domain for which it has better “known groups” validity than the Barthel index [6]. Thus, to assess for all the required domains using the SIS, the 59-item version, which is too long for routine use, would be necessary. Unlike the SIS-16 [6], the QLASS [8], and SASIP-30 [12], because the HRQOLISP-26 is comprehensive and covers all therapeutically relevant domains, it need not be combined with other measures to assess HRQOL in stroke patients.

 Therefore, in terms of striking a balance between content validity and brevity, the HRQOLISP-26 is better.

### 4.2. Precision, “Known Groups,” and Construct Validity

Precision is concerned with the number and accuracy of distinctions made by a measure, that is, precision of response categories or of numerical values [25, 26]. This is also indicated by the capacity of the measure to report the most favourable or poorest health states that is the paucity of floor or ceiling effects [25, 26]. The absence of significant ceiling and floor effects across the domains and overall scores in both countries coupled with the good construct and “known groups” validity as demonstrated by the Kruskal-Wallis statistics (Table  S2) [33] predicts good sensitivity to change [34, 35]. Typical of disease-specific measures tapping disease-specific concepts, it would be able to assess the worst and the best health states possible and detect small improvements and deteriorations [34, 35]. It would be useful in assessing the impact of therapeutic and rehabilitative interventions in stroke patients. However, prospective studies are needed to confirm this. 

Furthermore, in both settings, the HRQOLISP-26 was able to detect differences in HRQOL scores across mRS strata, particularly the physical and ecosocial domains to which it has similar construct. The overlaps in the boxplots for the cognitive and psychological domains by mRS strata is because the mRS is not a cognitive or psychological model. However, comparison of the HRQOLISP's psychological facet to corresponding SF-36 facets demonstrated good convergent validity [11].

Therefore, despite item reduction, the HRQOLISP-26 remains precise, and has good “known groups” and construct validity across its domains and as a whole.

### 4.3. Reliability

In both countries, the HRQOLISP-26 fulfilled Nunnally's criterion for internal consistency reliability with coefficients similar to those for SIS, SSQOL, and NEWSQOL and better than the SASIP-30 [12]. The conceptual model for its structure was corroborated by the fact that most domains fitted appreciably to a one-factor model [14, 20–22, 33, 36]. In both cities, the single-rater test-retest kappa statistics were excellent (>0.75) for most items (25 in Ibadan and 24 in Berlin) and good [24] for the remainder (Table 3) despite the slight difference in the timing of the repeat administration. Larger studies are required to assess its interrater reliability.

### 4.4. Brevity, Flexibility, Interpretability, and Acceptability

The HRQOLISP-26 can be completed within 7 minutes which saves 20 minutes compared to the HRQOLISP-102. This can be further shortened by selecting and combining only the domains that are appropriate to the design of a study [25, 26]. The possibility to combine the various domains of the HRQOLISP-26, which has been individually and collectively validated, is a unique flexibility of the HRQOLISP-26 which distinguishes it from other stroke-specific measures.

 Furthermore, unlike the SSQOL which is measured on a scale of 0 to 5 [7], the ordinal scale of 0 to 100 also improves interpretability. Therefore the unique brevity, flexibility, and interpretability of the HRQOLISP-26 would encourage its routine utility and acceptability in comparison to preexisting measures. This could be further improved by the ongoing development of a computer-based automated scoring and HRQOLISP wheel for prospective tracking of HRQOL in stroke patients.

### 4.5. Strengths, Limitations, and Future Directions

The HRQOLISP-26 is the shortest multidimensional, multiculturally generated and transnationally-validated, precise stroke-specific HRQOL measure. Although, in accordance with conventional practice [6, 7, 12], existing data was used in its development, and criterion validation [6, 7, 12], this is not a setback. This is because even though theoretically, actual answers on the HRQOLISP-26 may differ from answers retrieved for these items from the long HRQOLISP (as a patient's answers might be affected by other questions) [7]; concurrent application of the short and long versions is less feasible and may introduce the same bias that one wishes to avoid: influence on response by previous questions [7]. 

Notwithstanding, the HRQOLISP-26 is currently being used in a prospective international randomized control trial that would yield further information about its psychometric properties. Other prospective studies are also ongoing in different parts of Africa and Europe to further demonstrate its robust psychometric properties in diverse cultural settings. Also desirable are more extensive proxy-validation and additional external validation of the cognitive domain by cross-culturally validated cognitive scales which are applicable regardless of literacy level.

### 4.6. Conclusions and Implications

The HRQOLISP-26 is a novel, brief, multiculturally valid, and reliable stroke-specific measure. It is precise, interpretable, and flexible, comprising therapeutically relevant domains. It has good criterion, content, “known groups,” and construct validity. It is therefore recommended for routine clinical and research use.

## Supplementary Material

Participants included 103 stroke respondents (mean age = 66.9 *+* 11.6, male = 61) and 50 apparently healthy adults (AHA) from Berlin; and 100 stroke patients (mean age = 57.6 *+* 12.4, males = 41) with 100 AHAs from Ibadan.

## Figures and Tables

**Table 1:** Criterion validity of the HRQOLISP-26.

Berlin	HRQOLISP-63 mean, SD	HRQOLISP-26 mean, SD	Diff. in mean scores	Effect size of diff. in mean scores*	Mean diff. in individual scores (95% CI) between corresponding HRQOLISP-63 and HRQOLISP-26 domains^†^	Spearman rho^‡^	Explained variance of domain by short version (%)
Physical	65.1, 13.0	68.9, 17.5	−3.80	0.29	−3.91 (−5.26 to −2.56)	0.941^§^	88
Psychological	74.1, 12.3	69.4. 15.0	4.70	0.38	4.93 (4.01 to 5.85)	0.943^§^	92
Cognitive	75.5, 13.0	75.9, 14.1	−0.40	0.03	−0.47 (−1.39 to 0.45)	0.938^§^	91
Ecosocial	68.3, 9.1	68.6, 12.9	−0.30	0.03	−0.24 (−1.36 to 0.88)	0.917^§^	85
HRQOLISP-26	**70.8, 9.6**	**70.7, 12.2**	**0.10**	** 0.01**	**0.07 (−0.64 to 0.78)**	**0.968^§^**	**95**

Ibadan							
Physical	73.9, 14.1	72.5, 15.8	1.40	0.10	−0.01 (−1.83 to 1.81)	0.925^§^	92
Psychological	74.4, 13.5	69.0, 16.9	5.40	0.40	5.65 (4.42 to 6.88)	0.962^§^	92
Cognitive	71.7, 13.2	74.5, 15.4	−2.80	0.21	−2.91 (−4.26 to −1.56)	0.899^§^	82
Ecosocial	69.2, 10.1	71.2, 12.5	−2.00	0.20	−2.01 (−3.30 to −0.72)	0.916^§^	86
HRQOLISP-26	**71.3, 10.2**	**69.0, 14.1**	**2.30**	**0.23**	**1.42 (−0.60 to 3.44)**	**0.961^§^**	**93**

*Calculated by dividing difference in mean by SD of HRQOL-63^7^.

^†^Obtained by substracting HRQOLISP-26 score from HRQOLISP-63 scores.

^‡^Correlation between HRQOLISP-26 and corresponding HRQOLISP-63 domains.

^§^ *P* < 0.000001.

**Table 2:** Construct validity statistics for HRQOLISP-26 in both countries.

	Comparison between stroke and control groups					Kruskal-Wallis statistics of HRQOL		
						scores across stroke strata		
Berlin	HRQOL scores in stroke patients mean, SD *n* = 103	HRQOL scores in controls mean, SD *n* = 50	*t *values	two-tailed *P* values	ANCOVA (adjusted for age, gender, and SEC) *F*, *P* values	SLS strata *K-W * ^†^ **χ*^2^, * *P* values	mRS *K-W * ^†^ **χ*^2^, * *P* values	NIHSS strata *K-W * ^†^ **χ*^2^, * *P* values
Physical domain	68.99, 17.49	93.37, 3.86	−9.729	0.00*	25.759, 0.00*	44.4, 0.00*	69.2, 0.00*	54.4, 0.00*
Psychological domain	69.35, 14.97	82.45, 10.98	−5.504	0.00*	8.443, 0.00*	16.1, 0.00*	16.3, 0.003	19.6, 0.00*
Cognitive domain	75.98, 15.07	84.16, 9.70	−3.498	0.001	3.877, 0.005	5.54, 0.063	8.3, 0.080	6.7, 0.034
Ecosocial domain	68.64, 12.92	84.03, 8.33	−7.675	0.00*	22.453, 0.00*	18.1, 0.00*	39.2, 0.00*	30.3, 0.00
HRQOLISP-26	**70.74, 12.17**	**86.00, 6.49**	**−8.301**	0.00*	19.102, 0.00*	28.2, 0.00*	44.3, 0.00*	36.3, 0.00*

Ibadan	*n* = 100	*n* = 100						
Physical domain	72.5, 15.8	91.3, 7.6	−8.19	0.00*	10.443, 0.00*	18.9, 0.042	23.2, 0.00*	
Psychological domain	69.0, 16.9	81.6, 11.4	−5.55	0.00*	5.768, 0.001	28.0, 0.003	21.7, 0.005	
Cognitive domain	74.5, 15.4	84.0, 11.8	−4.69	0.00*	6.085, 0.001	26.9, 0.005	27.8, 0.00*	
Ecosocial domain	71.2, 12.5	78.2, 9.6	−3.931	0.00*	6.840, 0.00*	23.5, 0.015	25.0, 0.00*	
HRQOLISP-26	**68.9, 14.1**	**81.6, 8.4**	**−4.677**	0.00*	**5.487, 0.024**	**6.8, 0.03**	**12.0, 0.003**	

*P < 0.00001, *K-W* ^†^ *: Kruskal-Wallis test.*

SEC: socioeconomic class was computed using an aggregate of 3 variables (educational level, occupational strata, and average income).

NIHSS: National Institute of Health Stroke Scale.

SLS: stroke levity score, mRS: modified Rankin scale.

**Table 3:** Reliability statistics of the HRQOLISP-26.

Berlin	Number of items in original version	Number of items in shorter version (SV)	Percent explanation of SV by 1-factor solution	KMO, Bartlett *P*	Floor, ceiling effects* (domains)	Weighted kappa test-retest^†^ (items)	Cronbach's alpha for shorter version
Physical domain	16	7	55	0.83, 0.000	0,10	1.00	0.82
Psychological domain	12	7	54	0.85, 0.000	0,4	1.00	0.81
Cognitive domain	12	5	59	0.75, 0.000	0,8	0.71–1.00	0.82
Ecosocial domain	23	7	41	0.73, 0.000	0,3	0.69–1.00	0.76
HRQOLISP-26	**63**	**26**	**65**	**0.76, 0.000**	**0,3**	**0.69–1.00**	**0.81**

Ibadan							
Physical domain	16	7	50	0.74, 0.000	0,29	0.86–1.00	0.81
Psychological domain	12	7	52	0.83, 0.000	0,12	0.67–1.00	0.84
Cognitive domain	12	5	62	0.73, 0.000	0,20	0.76–1.00	0.84
Ecosocial domain	23	7	39	0.67, 0.000	0,8	0.85–1.000	0.70
HRQOLISP-26	**63**	**26**	**75**	**0.76, 0.000**	**0,8**	**0.67–1.00**	**0.89**

*Percentage of respondents with scores below 10% (floor) and above 90% (ceiling).

^†^Only 1 item had weighted kappa <0.75 in Ibadan, 2 items had weighted kappa <0.75 in Berlin.
