# Supplementary material for: HRQOLISP-26: A Concise, Multiculturally Valid, Multidimensional, Flexible, and Reliable Stroke-Specific Measure
Source: ISRN Neurol. 2011 Dec 6;2011:295096. doi: 10.5402/2011/295096 (PMC3263553; doi:10.5402/2011/295096)
Supplement: Supplementary file 1 — Participants included 103 stroke respondents (mean age = 66.9 + 11.6, male = 61) and 50 apparently healthy adults (AHA) from Berlin; and 100 stroke patients (mean age = 57.6 + 12.4, males = 41) with 100 AHAs from Ibadan. [file 295096.f1.pdf]

TABLE S1: Sociodemographic and clinical characteristics

	BERLIN		IBADAN	
Variable	Stroke group n= 103	Stroke group n=50	Stroke group n(%)=100	Control group n(%) = 100
**Gender**				
Male	61	11	41	41
Female	42	39	59	59
**Age, yrs**				
Mean (SD)	66.9 (11.6)	65.7 (5.9)	57.6 (12.4)	59.4 (9.9)
**Stroke type**	(CT/MRI)		(clinical)	
Ischemic	80		30	
Hemorrhagic	11		23	
Indeterminate/Mixed	9 (mixed)		47	
**Modified Rankin Scale**				
No symptom	4		0	
No significant disability	3		16	
Slight disability	34		27	
Moderate disability	24		24	
Moderately severe disability	8		31	
Severe disability	27		2	

FIGURE S1:Bland-Altman plots of the HRQOLISP-26 vs HRQOLISP-63 (Ibadan)

A: Physical domain Ibadan

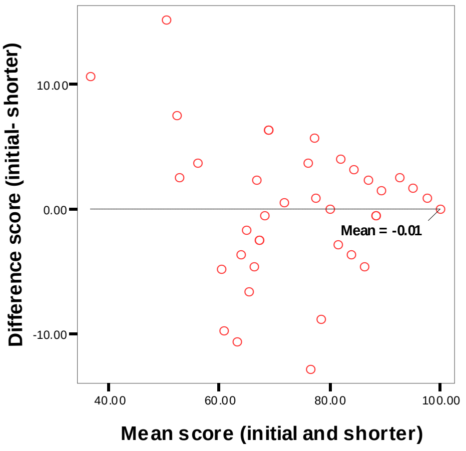

B: Psychological domain Ibadan

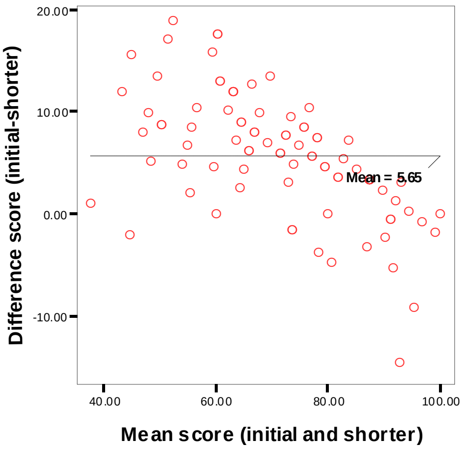

C: Total HRQOLISP-26 Ibadan

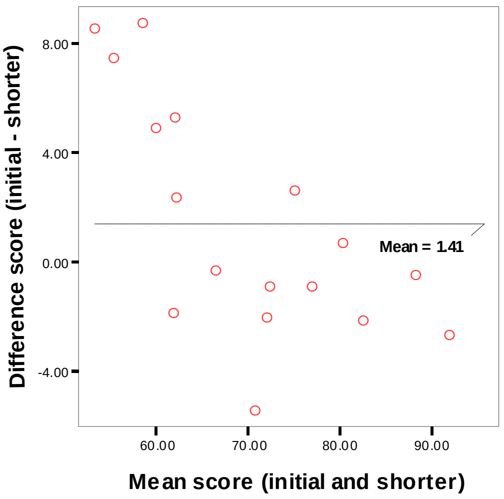

D: Cognitive domain Ibadan

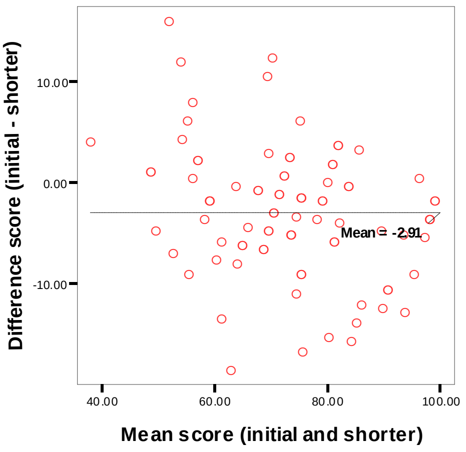

E:Ecosocial domain Ibadan

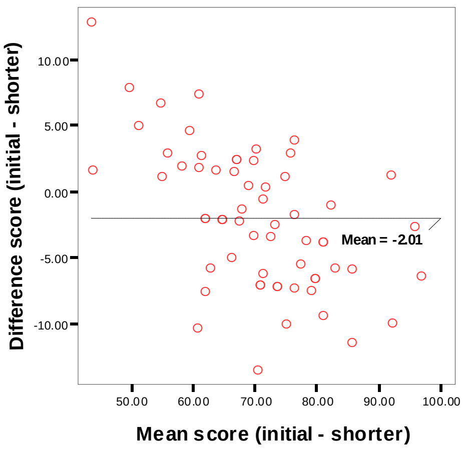

Bland-Altman plots of the differences between the 63-item physical sphere of the HRQOLISP (*initial*) and the HRQOLISP-26 (*shorter*) scores related to the mean of the HRQOLISP-63 and HRQOLISP-26 scores for domains and total score in Ibadan.

The *x* axes represent levels of HRQOL calculated as the means of the initial and shorter version scores and the *y* axes represent the differences between scores on initial and shorter versions. The horizontal lines represent the mean difference.

FIGURE S2: Box plot for HRQOLISP-26 scores versus mRS strata in Berlin

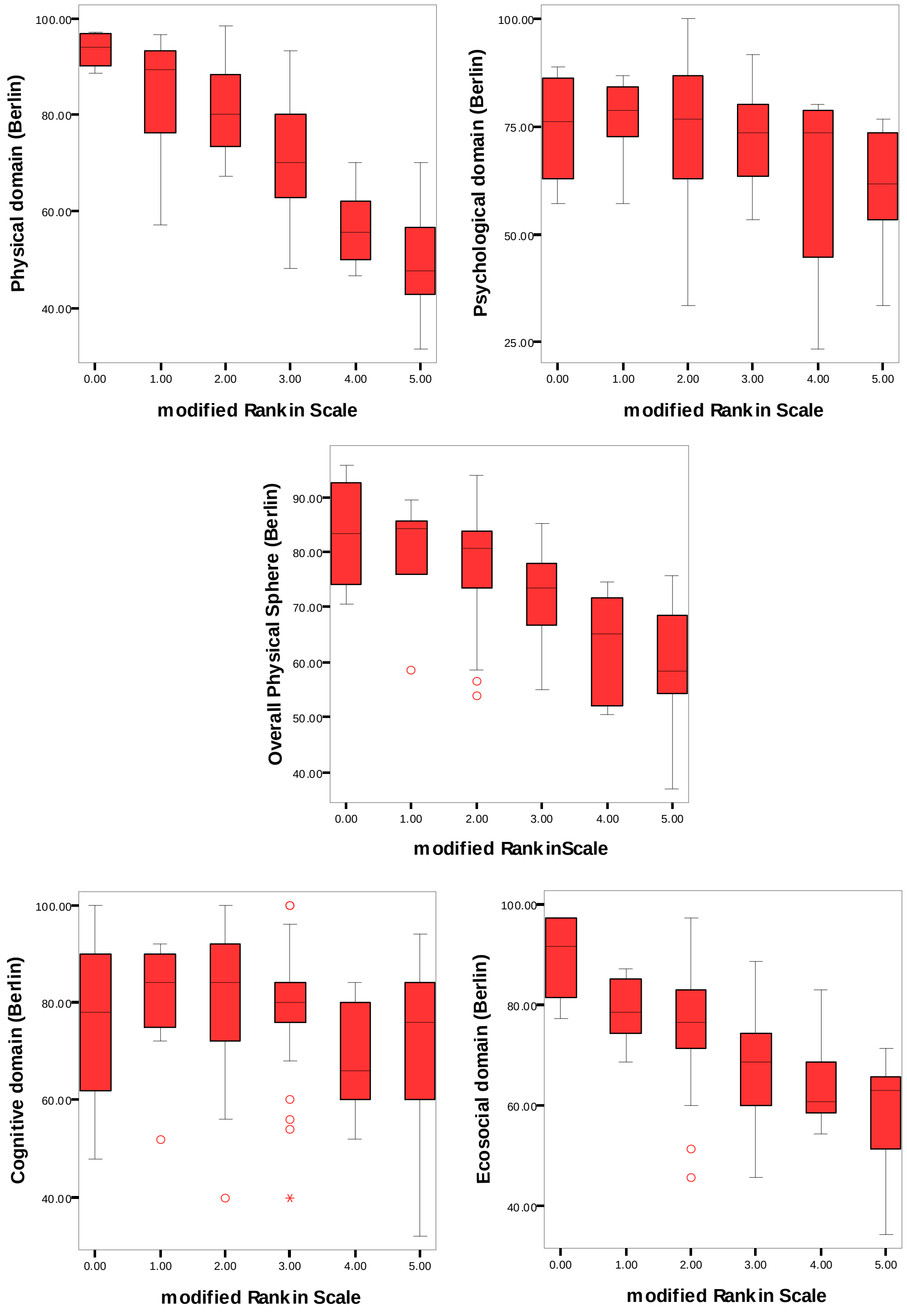

Appendix I : HRQOLISP-26 : The Health-Related Quality of Life In Stroke Patients (HRQOLISP) Questionnaire

INSTRUCTIONS

This assessment asks about how you perceive your current state of health, quality of life, or other areas of your life. **Please answer all the questions honestly**. If you are unsure about what response to give to a question, please choose the nearest most appropriate response. **Please keep in mind your standards, hopes, pleasures and concerns. Think about your life in the last two weeks. You should circle the number that best fits your response.**

	**1. PHYSICAL DOMAIN**					
1	Mobility	bed bound 1	chair bound 2	walks with helpers 3	walks with aids (frame/ tripod) 4	walks unaided 5
		**Not at all**	**A little**	**A moderate amount**	**Very much**	**Extremely**
2	To what extent do you have difficulties using your hands (eg gripping objects, turning door-knob, using cutlery, writing, opening jar/can, carrying heavy objects)?	0	1’	2’	3’	4’
3	To what extent do you have difficulties sitting/standing without losing your balance?	0	1’	2’	3’	4’
4	To what extent do you think physical pain or discomfort disturbs you?	0	1’	2’	3’	4’
		**Very dissatisfied**	**Dissatisfied**	**Neither satisfied nor dissatisfied**	**Satisfied**	**Very satisfied**
5	How satisfied are you with your ability to perform your daily living activities (feeding, bathing, toileting, dressing, grooming, e.t.c.)?	1	2	3	4	5
6	How satisfied are you with your capacity for work?	1	2	3	4	5
7	How satisfied are you with your sex life?	1	2	3	4	5

	**2. PSYCHO-EMOTIONAL DOMAIN**	**Not at all/ Never**	**A little/ Seldom**	**Moderately/ Quite often**	**Mostly/ Very often**	**Completely/Always**
**1**	How often do you have negative feelings such as blue mood, anger, despair, anxiety, depression, fear?	0	1’	2’	3’	4’
**2**	Do you have enough energy for everyday life?	1	2	3	4	5
**3**	To what extent are you able to accept your bodily appearance?	1	2	3	4	5
**4**	To what extent do you enjoy your work?	1	2	3	4	5
**5**	How often do you laugh?	1	2	3	4	5
**6**	To what extent do you enjoy your leisure?	1	2	3	4	5
**7**	How satisfied are you with your feelings?	**Very dissatisfied 1**	**Dissatisfied 2**	**Neither satisfied nor dissatisfied 3**	**Satisfied 4**	**Very satisfied 5**

	**3. COGNITIVE DOMAIN**	**Not at all**	**A little**	**Moderately**	**Very much/ Mostly**	**Extremely /Completely**
**1**	How well are you able to concentrate?	1	2	3	4	5
**2**	How available to you is the information that you need for your day-to-day life?	1	2	3	4	5
**3**	To what extent are you able to communicate?	1	2	3	4	5
		**Very dissatisfied**	**Dissatisfied**	**Neither satisfied nor dissatisfied**	**Satisfied**	**Very satisfied**
**4**	How satisfied are you with your ability to communicate?	1	2	3	4	5
**5**	How satisfied are you with your ability to think and learn?	1	2	3	4	5

	**4. ECO-SOCIAL DOMAIN**					
**1**	Activities of daily living(feeding, bathing, toileting, etc)	Fully dependent **1**	Requires substantial help **2**	Requires minimal help **3**	Requires no help but not back to work **4**	Back to work **5**
		**Not at all**	**A little**	**Moderately**	**Very much**	**Extremely/ Completely**
**2**	How much respect do you get from others?	1	2	3	4	5
**3**	How well are you able to manage your home and perform your domestic roles?	1	2	3	4	5
**4**	To what extent do you have access to transport facilities?	1	2	3	4	5
		**Very dissatisfied**	**Dissatisfied**	**Neither satisfied nor dissatisfied?**	**Satisfied**	**Very satisfied**
**5**	How satisfied are you with your personal relationships?	1	2	3	4	5
**6**	How satisfied are you with the support you get from your friends?	1	2	3	4	5
**7**	How satisfied are you with your access to health services?	1	2	3	4	5

Did someone help you to complete this form? **No** **1** interviewer **2** proxy

How long did it take you to complete this form ? __________minutes

Do you have any comments about this assessment?____

____

____

Appendix II : HRQOLISP-26 (German version)

INSTRUKTION

Diese Untersuchung fragt Sie wie Sie Ihren gegenwärtigen Zustand von Gesundheit, Lebensqualität und anderen Lebensbereichen wahrnehmen. Bitte beantworten Sie diese Fragen ehrlich. Wenn Sie sich einer bestimmten Antwort unsicher sind, wählen Sie bitte die am ehesten geeignete Antwort aus. **Bitte denken Sie an Ihre Maßstäbe, Hoffnungen, Freuden und Besorgnisse. Denken Sie an Ihr Leben in den letzten zwei Wochen. Kreisen Sie bitte die Nummer ein, die zu Ihrer Antwort am besten passt.**

	**1. PHYSISCHER ZUSTAND**					
1.	**Mobilität**	bettlägerig 1	rollstuhlmobil 2	gehen mit Hilfe 3	gehen mit Hilfsmittel (Stock/ Rollator) 4	gehen ohne Hilfe 5
2	Inwieweit haben Sie Schwierigkeiten Ihre Hände zu benutzen (z. B. zu schreiben, Dinge zu tragen)?	**gar nicht 1**	**ein bißchen 2**	**einiger maßen 3**	**sehr 4**	**außerordentlich 5**
3	Inwieweit haben Sie Schwierigkeiten Sie sich zu setzen/hinzustellen, ohne Ihr Gleichgewicht zu verlieren?	0	1	2	3	4
4	Inwieweit denken Sie dass physische Schmerzen/Beschwerden daran hindert das zu tun was Sie tun wollen?	0	1	2	3	4
5	Wie zufrieden sind Sie mit Ihrer Fähigkeit Tätigkeiten des täglichen Lebens auszuführen (z.B. essen, baden, Körperpflege, Toilettengänge, sich ankleiden, sich zurechtmachen, usw)?	**sehr unzufrieden 1**	**unzufrieden 2**	**weder zufrieden noch unzufrieden 3**	**zufrieden 4**	**sehr zufrieden 5**
6	Wie zufrieden sind Sie mit Ihrer Leistungsfähigkeit für Arbeit?	1	2	3	4	5
7	Wie zufrieden sind Sie mit Ihrem Sexualleben?	1	2	3	4	5

	**2. EMOTION/PSYCHE**	**gar nicht/ nie 1**	**ein bißchen /selten 2**	**einiger maßen/ ziemlich oft 3**	**meistens/ sehr oft 4**	**völlig/ immer 5**
**1**	Wie oft haben Sie negative Gefühle z. B. niedergeschlagenes Gefühl, Zorn, Verzweiflung, Sorge, Depression, Angst?	0	1	2	3	4
**2**	Haben Sie genug Energie für das tägliche Leben?	1	2	3	4	5
**3**	Inwieweit akzeptieren Sie Ihre körperliche Erscheinung?	1	2	3	4	5
**4**	Inwieweit haben Sie Freude an Ihrer Arbeit?	1	2	3	4	5
**5**	Wie oft lachen Sie?	1	2	3	4	5
**6**	Inwieweit genießen Sie Ihre Entspannung?	1	2	3	4	5
**7**	Wie zufrieden sind Sie mit Ihren Gefühlen? 1	2	3	4	5	

	**3. KOGNITIVER ZUSTAND**	**gar nicht/ nie 1**	**ein bißchen /selten 2**	**einiger maßen/ ziemlich oft 3**	**am meistens/ sehr oft 4**	**völlig/ immer 5**
**1**	Inwieweit können Sie sich konzentrieren?	1	2	3	4	5
**2**	Wie verfügbar für Sie ist die Information, die Sie brauchen für Ihr tägliches Leben?	1	2	3	4	5
**3**	Inwieweit können Sie kommunizieren (verstehen und Sie sich verständigen)?	1	2	3	4	5
**4**	Wie zufrieden sind Sie mit Ihrer Kommunikationsfähigkeit?	1	2	3	4	5
**5**	Wie zufrieden sind Sie mit Ihrer Denk- und Lernfähigkeit?	1	2	3	4	5

	**4. GESSELLSCHAFTLICHES LEBEN/ SOZIALE INTERAKTION**					
**1**	Tätigkeiten des täglichen Lebens (Essen, Körperpflegen, Toilettengänge, usw)	völlig abhängig **1**	erhebliche Hilfe benötigt **2**	ein bißchen Hilfe benötigt **3**	benötigt keine Hilfe, aber keine Berufstätigkeit **4**	wieder berufstätig **5**
**2**	Wie viel Respekt bekommen Sie von anderen Leuten?	1	2	3	4	5
**3**	Inwieweit können Sie Ihren Haushalt führen und Ihre häusliche Rolle spielen?	1	2	3	4	5
**4**	Inwieweit sind Verkehrsmittel für Sie zugänglich?	1	2	3	4	5
**5**	Wie zufrieden sind Sie mit Ihren persönlichen Beziehungen?	**sehr unzufrieden 1**	**unzufrieden 2**	**weder zufrieden noch unzufrieden 3**	**zufrieden 4**	**sehr zufrieden 5**
**6**	Wie zufrieden sind Sie mit der Unterstützung, die Sie von Ihren Freund/Innen bekommen?	1	2	3	4	5
**7**	Wie zufrieden sind Sie mit Ihrem Zugang zu Gesundheitsdiensten?	1	2	3	4	5

Hat jemand Ihnen geholfen diesen Fragenbogen auszufüllen
1 Interviewer/in **2** Angehöriger

Wie lange hat es gedauert den Fragenbogen auszufüllen? _________ Minuten

Haben Sie Kommentare zu dieser Untersuchung?

____

____

____

____
